# Heredity

**Published:** 1892-02

**Authors:** A. O. Rawls


### Heredity.

BY A. O. RAWLS, D.D.S.

From a scientific standpoint the study of Heredity virtually includes that of life in all its forms, from the most simple or lowest order to that of the highest and most complex organization. It embraces not only all of biology but adds other problems such as diversion or variation from typical forms, and reversion to earlier or remote ancestors, progenitors, etc.; in brief it includes the origin of life and species.

While an elaborate and detailed consideration of the various phenomena appertaining to this subject in its entirety as understood by numerous scientists, from the days of Lemarck and Darwin, down to the present time with its Spencers, Tyndalls and Huxleys, might not be uninteresting, yet the necessary scope of such consideration would preclude its appropriateness before this body. Some few among the world's recognized lights have delved their lives out to accomplish that which they have upon this subject, but the key which would unlock their store-house of knowledge is not made in a day.

W. K. Brooks, in his "Law of Heredity", opens the first chapter with the following statement, to wit, that "to the ordinary un-scientific reader the word heredity may perhaps suggest nothing more than a few curious cases where an odd peculiarity of the parent has been transmitted to the children, or it may recall the hereditary transmission of a tendency to certain diseases, or the mental or moral idiosyncrasies of the parents."

This is true if we take for granted his statement that a few curious cases when an odd peculiarity of the parent has been transmitted to the children, means many, and are of a physical character, and he surely means this, for his statement relative to the transmission of mental and moral idiosyncrasies includes all else transmissible.

The foregoing conceptions of heredity by the common mind or unscientific reader are virtually termed by scientists "odd cases, the tricks and accidents of heredity." Now while it is with these tricks and accidents of life's variation we are the more cognizant, and should bind our efforts, looking backward for a solution of, I cannot help but animadvert to the ideas of past research, which would term to-day's changes, tricks, and accidents of heredity of progenitors and environment, and associate the earliest known specialization of life with anything else but what such research has pleased to name, "Tricks and accidents."

They are results of such common observation indeed as to be recognized by the un-scientific mind, but nevertheless based upon laws as absolute and unswerving as are any other results growing out of natural selection and environment.

As progeny of the past, we have received the sum of impress, mentally and physically of countless ages, and our children will receive the same, added to which will be the effects of a weakened or strengthened, or in any wise changed condition of demand upon special organs or functions growing out of the hidden tendencies to variation of their immediate ancestors and environment differing from that of the latter. Since writing the above,

I find in W. K. Brooks work on "Heredity", chapter, "Evidence from Variation," the following, to wit: "We can also understand how a tendency to vary may be hereditary, for if certain cells of the body vary, they will exercise a disturbing effect upon adjacent or related cells, and these transmitting gemmules will hand on the tendency to vary to succeeding generations."

In his recapitulation and conclusion, Prof. Brooks has given us an additional statement in close association with the leading idea in the above, viz., that "There are many reasons for believing that variations under nature may not be so minute as Darwin supposes, but that evolution may take place by jumps or saltations, and that according to his view a change in one part will disturb the harmony of related parts, and will cause their cells to throw off gemmules. A slight change in one generation may thus become in following generations, a very considerable modification, and there is no reason why natural selection should not be occasionally presented with great and important saltations.

These latter statements are in perfect consonance with views which I have expressed at different times in the past, relative to the comparatively rapid transmission of the results of impress by certain influences upon various organs, to the generation in which they originated. If true, that most authority upon the subject of biology recognize the fact that variability and environment operate so slowly, that ages upon ages are required to envolve from generic types very marked changes of organisms as to form and functions.

Probably the most prolific animal known to us, and therefore the best suited for experiment looking to changes of form in their organization, is the white mouse.

By selecting from a litter of the latter a male and female, the largest and strongest of the lot and removing the external ear and tail, breeding them, and continuing to select the healthiest and best from each continuous breeding, and depriving each of ear and tail, you can have at the end of about eighty breedings, mice without the caudal appendage and external ear.

The Jewish nation evidently envolved the hooked nose, so common to that race, through their form of salutation, that is by taking the end of that organ between thumb and finger, and bowing their heads and bodies forward and downward. The Celtic race probably acquired the short and turned up nose by the constant wiping that organ upward with the palm of the hand.

The Indian and other wild tribes have kept up the inheritance from the generic type of pigeon toes, by turning their toes inward, the better to balance their stooping bodies while in pursuit of game or hiding from their foes.

The Mongolians under subjugation to the Tartars were made to train their hair up from the temples, and fasten it tightly over the top of their heads, which became a fixed habit and resulted in time in obliquity of their eyes.

The evidences herein adduced for the purpose of presenting an idea of how external organs may be caused to vary, or conformation to change by habit, custom, and environment, either forced or of free selection, are, I admit, by no means complete, but sufficient I trust to indicate the possibilities in this direction. Some one, however, may feel like exclaiming with the itinerant dentist from the vicinity of Jamestown or Canada, who had dropped in at a meeting of the American, at Niagara, and sitting in the back part of the room, had been listening attentively to scientific sentences, unabridged, from the lips of J. Foster Flagg, or our lamented Atkinson, when upon looking at his watch he innocently remarked to a neighbor, apparently of the same convictions and species, "Wonder when they'll begin on dentistry."

W. K. Brooks, "Evidence on variability from intellectual differences;" says, "That human advancement is, of course, widely different from the slow progress of the lower forms of life, but is fundamentally the same." In his closing chapter, also the following: "According to our theory of heredity, a change in one part of the body is in itself a cause of variation in related parts, and as changes thus tend to occur where and when they are needed, the time which is demanded for the evolution of a complicated organ by natural selection, is brought within reasonable limits, and one of the most fundamental objections is thus completely removed."

Now, if this be true, and we doubt not it is, for we believe Prof. Brooks is the best exponent of past research on the subject, and the most thorough reasoner extant, on its intricacies, then does it not bacome our duty, as specialists, laboring in the field of conservation of important organs, for those of our present generation, to have in view also an improvement of the form, structure, and health of such in coming generations.

A study, for instance, of any undesirable physical peculiarity of teeth, jaws and face of a family, the first, second and third generation of which are usually within the scope of our observation, will not only enable us to practice that which comes within the pale of our calling with more certainty of scientific results, but if we would live and labor not alone for those about us, I believe we can be the means of modifying the tendency of our generation to transmit its unhealthy variability in tissue, or disagreeable conformation of organs, and thus hand down to future generations a better basis from which to continue future labor in the line of health, beauty and usefulness until the type is near perfection.

Day by day we are brought face to face with an inheritance of dissease tissue, abnormal organization, and therefore disturbed function. With a fair knowledge of the laws of heredity and peculiarities sometimes attending family and racial proclivities and variation, how much better would the dentist be qualified to intelligently, and satisfactorily handle such conditions, indeed with how much more certainty could he diagnose the character of disease, and pronostigate the results of an operation.

Discussion on the papers of Wright, Harrison and Rawls.

*Dr. C. M. Wright.*—I have nothing particular to say on these papers. I was very much interested in Dr. Rawl's paper; such papers are always valuable. They present subjects that we cannot help be interested in, not only theoretically but practically, in our daily work. I do not wish at this time to discuss the question of heredity or the subject matter of any of the papers that have been read, including my own, which comes up for discussion at the present time.

*Dr. J. Taft*:—I only wish to say in regard to the three papers that have been presented to this body that I doubt whether there is any man present who is in a condition, merely from hearing the papers, to do himself justice in their discussion. They ought to be in the hands of the parties who are to discuss them long enough to consider and digest the salient points of each, and without this there can be very little in the way of discussion of either of these papers that will do justice to the writers, the persons who discuss them, or those who hear.

The method that is obtaining now in many societies of preparing papers and either sending a synopsis, or the whole paper preferably, to those who are to open the discussion, is eminently a desirable one, and it ought to be adopted by this society. Where a paper is carefully perused before-hand by those who are likely to participate in the discussion they can take up the subject and discuss it intelligently. The method of simply talking, for the sake of spending time, in a rambling way without any system is certainly not very profitable. It cultivates a faulty method. The society would do well to revise its method in this respect in the future, and I trust that may be done.

*Dr. C. H. Harroun*:—There is one point in Dr. Rawl's paper that I desire to call attention to, and that is with reference to the Indian walking pigeon-toed. I was brought up in near proximity with two large Indian camps in my early days and was very familiar with their habits, as were also my brothers who live in California, and who are conversant with the manner in which the Digger Indians climb mountains. If you wish to know why an Indian walks that way he will put his thumb down and ask whether you can walk on it. He turns in his toes to get the force of the whole foot. He does not walk on one organ, but turns his foot to get all the toes to strike so as to give him force. By so doing he travels better and easier.

*Dr. Otto Arnold*, Columbus. There were one or two points that occurred to me during the reading of these papers that I think ought not to pass without some discussion. They are points which I would like to have some light on, and they may have been observed by others. Dr. Harrison spoke of a general weakness of the whole system being always present where there are decayed teeth, or that they are always found together more or less. For instance, curvature of the spine is sometimes found in connection with poor teeth. In the few cases of that affliction that I have had occasion to work for, I found that the teeth were decidedly firm in texture. I would like to know if anyone else has noticed the same thing.

*Dr. J. R. Callahan*, Cincinnati. I was deeply interested in the paper read by Dr. Smith, and have been interested in this subject, more or less, for some time. In the first place, he spoke of the use of carbolic acid, of getting better effects from it, provided it was left in the cavity for several days. It has never been clear to my mind why it was better in a tooth a week than it was the moment it was put in. When it comes in contact with the albumen it coagulates it. If I rightly understand it coagulated albumen is insoluble and will remain in that condition for an indefinite time in the presence of the coagulent. I do not see how carbolic acid can go any farther under such conditions.

He spoke of other antiseptics, with some of which I am not familiar. He referred to terchloride of iodine. After reading Dr. Miller's article, some time ago, together with his experiments, I began to use it and have been much pleased with it. After the terchloride has been applied to the cavity and left in contact a few minutes the carious dentine takes on the color of iodine, or the diseased dentine seems to be saturated with the iodine. I have great faith in terchloride of iodine.

A member: Does it not stain the teeth?

*Dr. Callahan:*—No, only the diseased dentine so far as I have been able to ascertain. There may be a slight acid reaction. You can overcome that easily enough.

A member: Is it an irritant?

*Dr. Callahan:*—I think not. I have applied it directly to exposed and inflamed pulps with good results.

*Dr. Otto Arnold:*—I have had some very excellent results from the use of pyoktanin, confining the use of the remedy to the posterior teeth. It is highly soluble in water. In one case which came under my care a molar, which was quite far back nearly out of sight, a devitalized tooth, soon after the applications of the pyoktanin were made it became a dark blue. I saw a tooth that had been filled and finished perhaps two months later, and it was decidedly lighter, and when I saw it later it was still lighter. I am in hopes that, possibly, the stain would disappear entirely in the course of time.

*Dr. Taft*:—I will ask Dr. Arnold if he attributed the improvement in color to elimination of the agent or to decomposition of it?

*Dr. Arnold*:—I am not prepared to answer that. There were no absorbents, it being a devitalized tooth, I do not see how it could be eliminated.

*Dr. Taft*:—Then there must have been either decomposition of the substance, or it combined with some element of the tooth that was lighter in color or that changed the color. It would be interesting to know which process gave that improvement in color.

*Dr. Arnold*:—I will say that the patient for whom I performed the operation has left the State and I am unable to trace the case further.

*Dr. H. T. Smith*, Cincinnati. I also wish to state a case in regard to the use of pyoktanin in the treatment of teeth. A temporary molar, five or six months after treatment and filling, became considerably lighter in color, and it was believed to be due to the absorption of the pyoktanin through the temporary molar. I have tried the action of chloride of gold upon the decalcified dentine under amalgam fillings, that is placing it in a dry cavity and evaporating again to dryness, then filling with amalgam. The union was much greater than that which takes place ordinarily between the filling and dentine. The principle is used, I believe, by Dr. Land in uniting amalgam to porcelain; placing the chloride of gold on porcelain and evaporating or burning it dry, thus getting union between the gold and porcelain. Chloride of gold stains a black color.

A member: Has it any antiseptic properties?

*Dr. Smith*:—I do not know that it has.

*Dr. H. A. Smith*:—I quite agree with Dr. Callahan in regard to the point he makes about carbolic acid, if used in full strength, that it causes coagulability of the albumen. That means, of course, hardening, and it would soon produce a layer which would resist further penetration. If I used carbolic acid alone in sterilizing a considerable mass of carious dentine I would not use it stronger than a 50 per cent. solution. It is only a question of time with the limited amount of septic matter which we usually find in carious teeth when the sterilizing effect would be produced. I stated in the paper I endeavored to modify the action of carbolic acid. That I would not use it except in connection with an oily antiseptic. That is a point worth noticing.

*Dr. G. P. Gray*:—I agree with the remarks which were made by Dr. Callahan from the fact that we all know what difficulty we have in dealing with a pulp that is very nearly exposed, that is, in a condition such as Dr. Smith has referred to where there is a thin lamina above it. We know in our practice how many fillings we have to remove. We find this condition, the pulp dead, and a thin lamina there. In a great many cases the teeth seem to decay from the inner portion outward. There is a breaking down inward, and, of course, this is liable to be because of the portion left not being sterilized.

Some of the gentlemen have referred to using the new remedy known as pyoktanin. A gentleman told me he had been using it for a year, and it has acted like a charm. I do not think that is sufficient time to judge of results from its use, for many teeth after being filled, become devitalized and form what is called a blind abscess. We know very many times that teeth have these blind abscesses, or at least, apparently so. When we open one of them there is very little, if any, soreness; it does not seem to give much trouble. We diagnose the case; we say the tooth is devitalized, and immediately after an abscess forms or comes to the surface. Of course, we have theories as to the cause of that. It is apparent to me that atmospheric pressure and draining the dentine of fluid, etc., have caused the abscess to develop.

You will excuse me for digressing slightly. In putting antiseptics into cavities the trouble with sterilization is that it is liable to cause devitalization of the pulp. That seems to be the trouble, and whether carbolic acid would not be the best agent rather than the essential oils, under such circumstances, is a question in the minds of some. The essential oils are more penetrating and are more likely to reach the pulp. The pulp of a tooth is so delicate that it has slight resistance; almost any thing will act as a foreign body. Carbolic acid is said to be more local in its action, and if there is a thin lamina it will penetrate sufficiently deep to sterilize it, as it forms a kind of eschar, and will not endanger the pulp.

*Dr. J. Taft*:—I think it is well to give a little caution in reference to this matter. The way in which this subject is approached oftentimes (but not by the paper that has been read) would lead us to believe that in the larger proportion of cases of decay where cavities are to be filled, more or less partially decomposed, disintegrated dentine is left for some reason. Now, in the great proportion of cases there ought not to be, even partially, disintegrated dentine left in a cavity to be sterilized. After all, the question may arise whether after removal of all disintegrated dentine or tissue, sterilization may not be useful in a certain sense. Both sides of that question are dealt with in Dr. Miller's work. In one place in his work I was led to conclude that there was very little, if any, penetration of organisms beyond the line of disintegration. In another place in his work he treats the subject in such a way as to lead to the conclusion that his belief was that organisms are capable of penetrating to a considerable depth beyond the line of decalcification or disintegration of tissue. But then, after all, whether it is left or not, it becomes a question how much we ought to rely upon sterilization. I do not believe that it is as important as many imagine, because teeth were filled and decay arrested for years and years before anybody thought of sterilization, and even decayed portions of dentine were left over a nearly exposed pulp and covered, and yet decay did not progress. I very much doubt if a cavity is thoroughly cleansed from debris, and a portion of partially decalcified dentine or tissue is left over a pulp, and the walls of the cavity about the orifice are thoroughly dressed and a good filling introduced, between which and the walls of the cavity there will be no moisture admitted—that in very few such cases would decay take place. In years gone by there were multitudes of cases of this kind, made by good operators, and decay did not take place. It is perhaps less often than we think that cavities are perfectly sealed in a filling. I know in many cases we fail to make hermetically sealed cavities by filling. We may imagine that this has been accomplished thoroughly, all disintegrated tissue has been taken away and the filling introduced as thoroughly as possible, yet we know there are defective tracks through dentine that can not be perceived, that are not excavated, and teeth are filled irrespective of them. There will be openings sufficient to constitute a beginning of disintegration of tissue and thus penetrate into the tooth. Great care should be exercised in the preparation of cavities. I do not believe in leaving softened dentine in cavities ordinarily, only in favorable cases where the pulp would be quite exposed by its removal. In many cases it is better to take away all softened dentine about the orifice of exposure and trust to an artificial covering. I am reasonably certain that where the residuum is broken up, where it has lost its structural character, the probabilities are trouble will occur afterwards through irritation of the pulp. Therefore, in most cases, I take away the softened tissue, leaving none of it to act as an irritant to the pulp. I suppose one of the reasons why sterilization is resorted to by so many is to arrest decay, but after all we are led to conclude by the discussion that the great danger is, in the progress of the decay in the dentine beyond the filling. So I think great care should be exercised in forming cavities and introducing fillings as well as in sterilization. I fear we are too much inclined to leave cavities in an improper condition for filling, introducing fillings when there is disintegrated tissue that ought to be removed. If, however, a pulp is covered by tissue that is leathery in consistence and structure not wholly destroyed, it may be retained with a good hope of saving the pulp. I have oftentimes had my attention called to cases like this, where softened dentine was left in a cavity in considerable extent, and the cavity thoroughly sealed. Years after, when removed, the whole portion was as solid as a secondary deposit of dentine in the cavity. I presume that does not sufficiently frequently occur as to be relied upon as a mode of practice, but it does occur sometimes. I have seen it in cases which I have had in charge, and in cases in the hands of others. We should not put too much stress upon one particular of practice while we leave others without the attention they ought to have. That is simply the point I wish to emphasize in the matter. Let us give attention to every part in these operations. I know that fillings are made sometimes over softened dentine without sterilization, and decay does not go on. Whether decay does not sometimes go on after sterilization may be a question. I rather suspect it does. I have seen operations from the hands of those whom I knew were strong advocates of universal sterilization and I found decay going on beneath the fillings, but in no instance have I seen decay under a filling hermetically sealed and which remained so afterwards. I have yet to see a case of that kind. There may be such cases but I have not seen them.

*Dr. C. M. Wright:*—Such remarks as have just been made by Dr. Taft plunge me into despair, because if his opinions prevail, I see no hope of relief from the slavery of work. When I hear such papers as Dr. Smith has offered to-day I think we are in the right line; the little birds begin to sing about my heart of hope, hope that we shall not have to fill teeth with amalgam, and gold and tin and gutta-percha, and to use the dental engine, the chisel, the file, and the plugger, all the days of our life. The paper would lead us to hope for that time when dental practice will be confined to receiving patients in the most agreeable sort of manner, saying, "Madam, walk in; name, if you please; be seated." Our instruments will consist of small rods, little swabs, camel's hair pencils and drugs; when we can tell by the kind of debris the nature of the microbe, whether anærobic or ærobic, pathogenic or non-pathogenic, and select our remedy, pencil the part lightly, receive our fee and bid the madam good morning.

(Laughter). I have been waiting for this all these years. I was quite happy until Dr. Taft plunged me back into the same old rut, which means working from morning till night. (Applause).

> " From early chime to chime
> As prisoners work for crime."

I am so sorry!

*Dr. A. O. Rawls*, Kentucky. The question of antiseptics in dental practice has been touched upon and I regret I was not present to hear the whole of the paper. Prof. Smith referred to this matter as it concerned the sterilization of tissue or decomposed dentine. I apprehend that the gentlemen who have been talking on this subject have been delving a little bit deeper than they have knowledge of. In the first place, if we consider the question as it refers to decalcified dentine over a pulp, do we know from the gentleman's own statement this morning, supported by Dr. Wright, I believe, that there was no change in the tissue as to calcific matter and animal matter in the youth or aged. If we take it as we have been studying it from the standpoint of Dr. Taft's remarks, then we find that the nutrient canals or currents supply the dentine that is above and below and roundabout with life, etc. We find these canals open or closed, one of the two. Sterilization means this, that the particles or fluid you use for sterilization must penetrate, must go into the substance of that cavity or canal that has been deprived of its lime salts. Having been deprived of lime salts, therefore, there is a change of structure. This knocks out some of the arguments advanced by gentlemen this morning. My idea of sterilization is simply that you have to shut a thing up that is not septic—tissue that is not septic. Septic tissues decompose and disintegrate whether shut up or not. The paper claims that you use a substance that will penetrate or antisepticize tissue. What do you know about antiseptization in these cases? You can not see into it by the microscope. It is a matter of results. You can not tell whether you have complete or incomplete antisepticism. We closed up cavities before without a knowledge of antiseptics, and the results were just as good, and often a good deal better, because we have been making sick teeth by too much antiseptic treatment when we would not make them otherwise. But there is this much in that practice, that the substance that lies between the exposed, or would-be exposed nerve and the outer cavity has no organization virtually. It is simply a shell or skeleton of that which was, and it being a skeleton it exists as it is simply because of the integrity of this tissue. You do not want to sterilize that. If it has the integrity to stay there you do not want to sterilize it, but to protect it. There is nothing going on in a layer when it is dead and aseptic. I think that that condition of a tooth is just as plain as day, the same as that condition which would be presented by any tissue of the body of a bony nature; that is, if you have decalcification or removal of any special element or particle of that tissue without any outside influences that would conduce to its change, then all you have to do is to protect the integrity of that tissue so long as its integrity will last, until some thing outside attacks it. Professor Taft said there is a perfection, or a possibility of perfection in protecting this condition. That is a mistake. There is no such thing as perfect contact. There is no such thing as the absolute contact of a filling or nerve capping. There is no such thing as that in either dentine or enamel. Therefore, there must be some influences on the outside that work roundabout, and in between, when there is no contact. We can not have it otherwise. As Sir Isaac Newton, the great astronomer, said: "Compress the world into one solid mass and you can put it into two inches square." Where do you get absolute contact of a tooth? At any rate there is no such thing as having absolute contact of a filling or a capping material in such a manner that it will preclude the possibility of changes beneath and about it. We have the experience that a few years filling will conserve a certain portion of the dentine and enamel with comparative contact with it, but when it comes to *absolute* contact there is no such thing as that. That is just exactly what we have to contend with, and what we are mistaken about in our efforts to assist the continuance of the integrity of the tissue upon which we work. We bruise or change the condition of a surface by hammering, by the chemical influences of a substance which we put in it, otherwise we could protect and conserve by different means to those, but only for a time. As a rule antiseptics are not necessary in cases of deep decay, because, generally speaking, antiseptics have nothing in the shape of septic matter to operate against in deep-seated caries. That is simply a destruction of tissue by a lack of nutrition, and a lack of nutrition does not necessarily mean that there are septic matters there to cause this lack of nutrition.

*Dr. H. A. Smith*:—What about the micro-organisms there?

*Dr. Rawls*:—We do not know that they are always there.

*Dr. Smith*:—Yes. we do.

*Dr. Rawls*:—You can not prove that they are there beyond a certain depth without removing tissue of importance to the tooth, a certain place in the semi-disorganization or disintegration.

*Dr. Smith*:—There is no disorganization in this substance.

*Dr. Rawls*:—You said yourself there were no septic germs, and that you cleaned out the cavity down to a point where there was no septic matter.

*Dr. H. A. Smith*:—When I hear persons of the mental calibre of Prof. Taft and Dr. Rawls address a body like this with such remarks as they have made to-day, I soon discover that they do not believe in the chemico parasitical theory of decay. Dr. Taft does not believe that hypothesis, and hence he is not good authority when he discusses sterilization of carious dentine. If we can fix in our minds just what the layer is we are treating, how it is constituted, what changes have occurred in it, we can better understand the matter. I said we had in the upper layer a portion of softened dentine which was formerly the organic part of the tooth—the matrix. Next, we have a portion of softened dentine in which the micro-organisms are active in producing caries. They secrete lactic acid, which dissolves the dentine beyond the layer in which they are found. But for this chemical action, the softening of the dentine, the micro-organisms could not advance and caries would be arrested. If we accept this theory as true, then an antiseptic is essential to prevent further change in the organic (albuminous) portion which is left in the bottom of the cavity for protection of the pulp. Now, an antiseptic will do all this without endangering the pulp if it is judiciously applied. Then we have fixed matter, and until we do accomplish that, with all due deference to my friend on the left (meaning Dr. Taft) we have no assurance we can conserve teeth in which the pulps are nearly exposed.

He (Dr. Taft) refers to what was done formerly. I can go back a good way myself. We did not make a record of our cases then as we do now. We observe better now. When we fill a tooth now, if a pulp inflames and dies, we trace the cause. We did not do it formerly. There were hundreds of pulps that died under fillings. We do not have the trouble following filling now which we used to have. We recognize the sensitiveness of dentine; we interpose a non-conductor; we sterilize and all that, and our patients are much more comfortable than before. I know from my own experience that my patients do not come back to me after I fill their teeth with abscess, with the teeth subject to changes of heat and cold, etc. Improved methods in practice come from enlightenment or from the advancement that has been made in dental science. Therefore, I speak from that standpoint, and accept as the best explanation to-day of the etiology of dental caries, the presence of micro-organisms. I use a sterilizer for the purpose of arresting their life forces, changing the soil in which they live and thus wiping them out of existence. It is only on this theory of the explanation of decay that we treat these cases. If we do not accept it antiseptics are not necessary.

The same difference of opinion holds good in the practice of general surgery. There is an eminent man in England, whose name I can not at present recall, who pooh-poohs antisepticism (a member: Lawson Tait, of Birmingham, England) yet he is one of the cleanest men in his operations in the world. He is alone, we might say, in this regard. He does not recognize the need of antiseptics, and yet his operations are attended with great success. But Mr. Tait is an exception. The average surgeon who does not use antiseptics in his operations has dirty hands and finger-nails, and in a great many instances his cases are unsuccessful as far as results are concerned. It is the clean surgeons, those who adopt such antiseptic methods who are most successful in their operations. Asepsis means cleanliness, and cleanliness—no matter how accomplished—is asepsis. I differ with Professor Taft and prefer leaving the natural covering over a pulp undisturbed if I can prevent further change in it by sterilization. Whenever the pulp of a tooth is exposed to the atmosphere it is liable to become contaminated with septic matter and hence its preservation is endangered.

*Dr. Rawls*:—I may have been placed in a false position before this society on the suject of antiseptics. I did not mean to state that I was not in favor of all antisepticism that was of value to any condition or disease connected with the mouth that we deal with; at the same time I was simply arguing as to the possibilities of the effects in these cases, and not as to the possibilities of our knowledge, as to *how* or *why* they should produce these results, since we have not seen by the microscope any special difference between one treatment and another. Now there is only one means by which antiseptics can produce the result and that is by entering into the substance of the tissues.

A member: If they enter into the substance of the tissues do they become part of the tissues?

*Dr. Rawls*:—Yes, to a degree.

A member: Or are they absorbed by dead tissue?

*Dr. Rawls*:—To a certain extent they are, as carbolic acid or pyoktanin, either one of these substances will be taken up by the absorbent vessels and carried out through the system.

A member: Are they of an insoluble nature?

*Dr. Rawls*:—They must be insoluble. There must be a line of life; there must be an anastomosis between death and life, or a line of demarkation, and where you have dead tissue, what is the difference to you if that is shut up whether you sterilize or not? You can not say that the micro-organisms produce these results. You can not say that they were diseased, they died, and as a result of their death you say an acid was produced. You can not say that knowingly. You assume it. Therefore, we are working in the dark on the subject of antiseptics in a dead material that we want to save the integrity of. If you would preserve that tissue you put something in that does not belong there, you want protection, not antisepticism, of dead tissue.

*Dr. Taft*:—I want to say a few words in reply to the remarks of Dr. Smith. I would much prefer you would take my own statements as to what I believe, and the theories I espouse than to have him say what I believe, and what I do not believe. I am not so ready to make square assertions about things that have never been clearly demonstrated. I do not believe that there are organisms (vegetable organisms) in connection with all cases of decay of the teeth, and I have said this a great many times. They have some influence undoubtedly, but what that influence is, and exactly how it is operative, I do maintain has not been made clear as yet, hence much of the practice that is employed in this direction is experimental, not to say empirical. It is upon a certain hypothesis that has not been clearly demonstrated. Perhaps many of these organisms live without air (anærobic), and some of them can only live with air. Dr. Rawls is rather inclined to doubt perfect contact or perfect adaptation, which is in one sense true. A cavity sealed up so as to exclude all foreign substance, liquids, every thing of this kind, is hermetically sealed. I believe in the use of antiseptics, but then my use of them is not based upon the theory that antisepsis is a necessity in all cases; that has not been demonstrated.

*Dr. Smith*:—Do you refer to the etiology of caries now?

*Dr. Taft* (continuing):—Not so much that. I refer simply to the condition as it exists and is found. These organisms do sometimes penetrate dentine, but not deeply, however. I have a number of slides of dentine prepared in which it seems that they penetrated and seemed to be in the tubules beyond the line of disintegration and decalcification. One or two of these slides were prepared by Professor Miller.

Just what influence these micro-organisms have in the production of decay is not sufficiently proven. There are various theories on this point. We saw at Saratoga, last summer, a number of teeth in the mouth of a boy in which decay seemed to have been arrested by the use of nitrate of silver. The action of the caustic upon the tissue arrested the decay. This was done without any other antiseptic.

*Dr. Smith:*—Yes, it was the caustic action of the nitrate of silver which destroyed both the bacteria of caries and a superficial layer of dentine. The silver nitrate by forming an insoluble compound with the layer prevented the further action of caries.

*Dr. Taft* (continuing):—I would like to know as much about it as a great many claim to know. I have not got to it yet.

*Dr. H. A. Smith:*—We should accept the doctrine of Dr. Miller because we can produce precisely the same conditions out of the mouth that we find in carious teeth in the mouth. We can infect dentine out of the mouth and produce true dental caries, therefore we see that this is a plausible theory. I remember it is not many years ago when I was disposed not to accept the so-called mineral acid theory, and I was criticised, in this society, for being a little too advanced. Now it is generally believed that micro-organisms play a very important part indeed; that they are essential to the production of caries of the dentine. Dr. Taft appears to be one of the few who are not yet convinced.

